# Pivotal Role of Tenascin-W (-N) in Postnatal Incisor Growth and Periodontal Ligament Remodeling

**DOI:** 10.3389/fimmu.2020.608223

**Published:** 2021-01-22

**Authors:** Thomas Imhof, Anamaria Balic, Juliane Heilig, Ruth Chiquet-Ehrismann, Matthias Chiquet, Anja Niehoff, Bent Brachvogel, Irma Thesleff, Manuel Koch

**Affiliations:** ^1^ Faculty of Medicine and University Hospital Cologne, Institute for Dental Research and Oral Musculoskeletal Biology, University of Cologne, Cologne, Germany; ^2^ Center for Biochemistry, Faculty of Medicine and University Hospital Cologne, University of Cologne, Cologne, Germany; ^3^ Institute of Biotechnology, HiLIFE, University of Helsinki, Helsinki, Finland; ^4^ Cologne Center for Musculoskeletal Biomechanics, Faculty of Medicine and University Hospital Cologne, University of Cologne, Cologne, Germany; ^5^ Friedrich Miescher Institute for Biomedical Research, Novartis Res. Foundation, Basel, Switzerland; ^6^ Department of Orthodontics and Dentofacial Orthopedics, School of Dental Medicine, University of Bern, Bern, Switzerland; ^7^ Institute of Biomechanics and Orthopaedics, German Sport University Cologne, Cologne, Germany; ^8^ Department of Pediatrics and Adolescent Medicine, Experimental Neonatology, Faculty of Medicine and University Hospital Cologne, University of Cologne, Cologne, Germany; ^9^ Center for Molecular Medicine Cologne (CMMC), University of Cologne, Cologne, Germany

**Keywords:** tenascin-W, tenascin-N, bone, remodeling, periodontal ligament, pain, tenascin

## Abstract

The continuously growing mouse incisor provides a fascinating model for studying stem cell regulation and organ renewal. In the incisor, epithelial and mesenchymal stem cells assure lifelong tooth growth. The epithelial stem cells reside in a niche known as the cervical loop. Mesenchymal stem cells are located in the nearby apical neurovascular bundle and in the neural plexus. So far, little is known about extracellular cues that are controlling incisor stem cell renewal and guidance. The extracellular matrix protein tenascin-W, also known as tenascin-N (TNN), is expressed in the mesenchyme of the pulp and of the periodontal ligament of the incisor, and is closely associated with collagen 3 fibers. Here, we report for the first time the phenotype of tenascin-W/TNN deficient mice, which in a C57BL/6N background exhibit a reduced body weight and lifespan. We found major defects in the alveolar bone and periodontal ligament of the growing rodent incisors, whereas molars were not affected. The alveolar bone around the incisor was replaced by a dense scar-like connective tissue, enriched with newly formed nerve fibers likely leading to periodontal pain, less food intake and reduced body weight. Using soft food to reduce mechanical load on the incisor partially rescued the phenotype. *In situ* hybridization and Gli1 reporter mouse experiments revealed decreased hedgehog signaling in the incisor mesenchymal stem cell compartment, which coordinates the development of mesenchymal stem cell niche. These results indicate that TNN deficiency in mice affects periodontal remodeling and increases nerve fiber branching. Through periodontal pain the food intake is reduced and the incisor renewal and the neurovascular sonic hedgehog secretion rate are reduced. In conclusion, tenascin-W/TNN seems to have a primary function in rapid periodontal tissue remodeling and a secondary function in mechanosensation.

## Introduction

**Figure 1:**
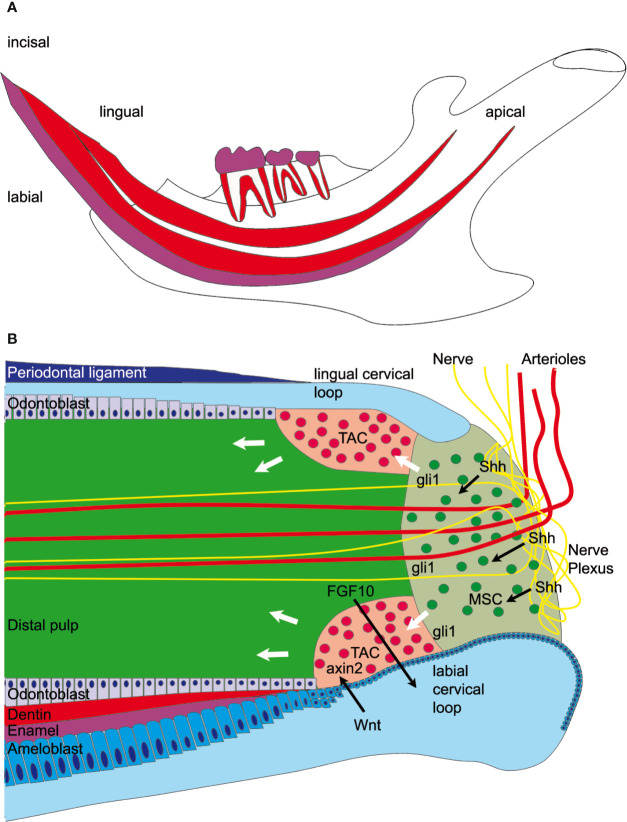
Graphical overview of the mandibular incisor anatomy. **(A)** A large fraction of the mouse incisor is embedded in the mandible. Enamel covers (magenta) only the labial surface of the tooth. Mesenchymal stem cells (MSC) and epithelial stem cells are found in the dorsal mandible. **(B)** Mesenchymal stem cells are regulated by sonic hedgehog (Shh) released from the neurovascular bundle and express Gli1, which is a hedgehog signaling effector. Those stem cells eventually proliferate in the transient amplifying cell (TAC) zone and are regulated by epithelial-derived Wnt. Axin2, a signaling molecule from the Wnt pathway, is a marker for those highly proliferative cells. The mesenchymal TAC cells then differentiate into odontoblasts and form dentin or are differentiating into pulp stroma cells. The epithelial stem cells are found in the labial cervical loop and they differentiate into ameloblasts and form enamel.

Rodent incisors, unlike human teeth, grow continuously throughout life. In young animals the entire length of the lower incisor renews every month (1). The incisor tip self-sharpens due to the asymmetrical distribution of the enamel, which covers only the labial surface of the tooth enabling abrasion of the softer dentin on the lingual surface resulting in a sharp labial enamel edge. Though the lower incisors are only visible at the most ventral aspect of the mandible, within the jaw itself the incisors occupy almost the entire length of the body of the mandible (**Figure 1A**). In the dorsal mandible, epithelial and mesenchymal stem cells reside in the incisor and they provide a continuous supply of hard tissue forming cells. The epithelial stem cell niche is morphologically clearly defined; it is localized in a loop-shaped ending of the epithelial layer called cervical loop (**Figure 1B**). Cervical loop is composed of outer and inner epithelia, which enclose loosely arranged stellate reticulum cells, including stem cells. The epithelial stem cells differentiate into enamel secreting ameloblasts. On the other hand, dentin is produced by odontoblasts, which derive from the incisor specific mesenchymal stem cells (MSCs) just recently discovered (2–4). Lineage tracing experiments showed that Schwann cell precursors, which express Sox10 and PLP1, generate a major population of dentin producing odontoblasts (3). A second, vasculature associated mesenchymal stem cell population includes slowly dividing population expressing Gli1, as well as aSMA and NG2 expressing cells (2, 4, 5). Gli1 expressing population is regulated by Hedgehog signaling which is transduced through two Hedgehog ligands; Shh and Dhh (4, 6). Proliferation and differentiation of tooth stem cells is directed toward the tip of the tooth and is controlled through epithelial-mesenchymal crosstalk (7). In recent years it became clear that extracellular matrix proteins have a key role in controlling the organization of cell compartments: they provide cell attachment sites, form barriers, present growth factors and control the mechanical properties of the tissue (8). Each tissue has an unique composition and topology of its extracellular matrix (9). The structure and function of enamel and dentin matrix proteins such as ameloblastin, amelogenin, dentin matrix protein, and dentin sialophosphoprotein are well studied. However, the microenvironmental factors that control pulp stem cell fate are less well known. In this context, tenascins are highly interesting as they are expressed in embryonic mesenchyme and stem cell niches (10). Tenascins are large multifunctional proteins, which have a common domain structure: an N-terminal assembly domain, epidermal growth factor (EGF)—like repeats, fibronectin type III domains, and a C-terminal fibrinogen-like globular domain. In mammals four tenascins are expressed: Tenascin-C, -R, -W/(-N), and –X. One well known function of tenascins is their ability to modulate cell adhesion and migration *in vitro* (11). Tenascin-C and -R bind to neural cell adhesion receptors and co-receptors such as contactin (12, 13), syndecan (14), and integrins (11). Tenascin-C is expressed in the central nervous system and in embryonic connective tissues. Mice deficient for tenascin-C were originally reported to develop normally (15), but more recently behavioral phenotypes and abnormal central nervous system development were described (16). The expression of tenascin-R is limited to the central nervous system (17) and the knockout mice show cognitive defects, reduced coordination and increased anxiety (18). In contrast, tenascin-X is widely expressed in loose connective tissues (19), and tenascin-X deficient mice show an Ehlers-Danlos syndrome-like phenotype with altered fibrillar collagen density and hyperextensible skin (20).

Tenascin-W, also known as tenascin-N, is widely expressed during embryonal development (21), but in adult mammals its expression is mainly restricted to the periodontal ligament (22), periosteum (21), and stem cell niches (10). In cancer, TNN expression is strongly induced and for breast cancer a role in migration and metastasis has been proposed (23, 24). *In vitro* experiments show that tenascin-W/TNN promotes migration of several cell types, including breast cancer cells, osteoblasts, and endothelial cells (23, 25, 26). Hence, TNN may play an important role in cell migration and differentiation in the continuously growing incisor tooth.

In this study, we have analyzed the role of tenascin-W/TNN using a global tenascin-W/TNN deficient mouse. This first analysis of tenascin-W/TNN deficient animals showed that tenascin-W/TNN has a function in incisor periodontal ligament remodeling, in incisor eruption and in tooth renewal. Tenascin-W/TNN deficient mice show under hard pellet diet weight loss and reduced food uptake.

## Materials and Methods

### Mouse Model

For the generation of tenascin-W/TNN knockout mice, the construct Tnn^tm1a(KOMP)Wtsi^ was purchased from the KOMP consortium. The mice were generated and the neo-cassette has been removed by crossing with a CMV-Cre mouse line. Heterozygous mice were backcrossed 12 times with C57BL/6N wildtype animals in a specific-pathogen-free animal facility. Genotyping was performed by PCR with the following primers: TNN(-/-) fw: 5´ ttcactgcattctagttgtgg 3´, TNN(-/-) rev: 5´ caggaagatcgaggatctggc 3´, TNN WT fw: 5´ catactcccatgcacacttcc 3´, TNN WT rev: 5´ ctttgcctctagaagtatggacc 3´. The animals were fed with γ-irradiated pellet or powder form diet (1310-recipe, Altromin) and wooden gnaw sticks were placed in all cages. Gli1CreER^T2^ x R26mTmG x TNN(-/-) mice were generated by crossing heterozygous Gli1CreER^T2^ mice (27), heterozygous R26mTmG mice (28), and TNN(-/-) and control mice without neo-cassette. Genotyping was performed by PCR. For Gli1CreER^T2^ the primers fw: 5´ acctgaagatgttcgcgattatct 3´, rev: 5´ accgtcagtacgtgagatatctt 3´; and for R26mTmG the primers fw: 5´ gtgagcaagggcgaggagctg 3´, rev: 5´ ttacttgtacagctcgtccatgc 3´ were used. TNN(-/-) and WT animals of both sexes were sacrificed by cervical dislocation (1mt, 3mt, 6mt, and 12mt) or decapitation (P0 and P7 old mice). 3 month old Gli1CreER^T2^ x R26mTmG x TNN(-/-) and Gli1CreER^T2^ x R26mTmG x control mice of both sexes (mice, n = 6) were treated twice every 24 h per i.p. injection with 10 mg of tamoxifen (T5648, Sigma) dissolved in corn oil (C8267, Sigma). The lineage traced animals were sacrificed by cervical dislocation 72 h after the first injection. All experimental procedures were approved by the State Office of North-Rhine Westfalia (AZ 84-02.04.2015.A454 and AZ 81-02.04.2018.A367).

### Histology

Hematoxylin-eosin (H&E) stainings, detection of endogenous alkaline phosphatase and Sirius red stainings were performed on 5 µm thick paraffin sections (mice, n = 6) as previously described (29, 30). For immunofluorescence staining tissues were dissected from animals (mice, n = 6), fixed with 4% paraformaldehyde (PFA) in PBS at 4°C overnight. After extensive washes in PBS, the samples were decalcified with 10% EDTA for 14 days under constant agitation, and with daily changes of the decalcification solution. The decalcified samples were then washed with PBS for at least 4 h, infiltrated with 30% sucrose/PBS, and embedded in OCT Compound (TissueTek, Sakura). From each animal (mice, n = 6) a comparable section was chosen for individual staining. Due the small size of this tissue, often only one perfect cryosection per mandible was obtained. Cryosections (40 µm) were permeabilized with 0.3% Triton/PBS for 2 h and blocked with 10% normal goat or donkey serum overnight at 4°C. Primary antibodies were diluted in 1% BSA/0.1% Tween/PBS and applied to the sections for 30 h at 4°C. They were: Tuj1 (ab18207, 1:1000, Abcam), CD31 (1 µg/ml, polyclonal from rabbit, own production), collagen 3 (1330-01, 1:400, Southern Biotech), Ki67 (ab16667, 1:500, Abcam), Sox10 (sc-17342, 1:500, Santa Cruz), ameloblastin (Ambn) (1 µg/ml, polyclonal from rabbit, own production), tenascin-W/TNN [1 µg/ml, polyclonal from rabbit (21)], GFP (SP3005P 3 µg/ml, Origene), and smooth muscle actin (C6198, 1:500, Sigma**)**. Alexa dye conjugated secondary goat or donkey antibodies (Thermo Fisher Scientific) were used to detect the specific binding. Confocal images were obtained on a Leica SP8 microscope, using a 25x water objective or a 20x air objective. The images were processed with ImageJ 1.51r software (31) and brightness and contrast were adjusted.

### *In Situ* Hybridization

Radioactive *in situ* hybridization was done on 5 µm sagittal sections (32). ^35^S (Amersham)-labeled RNA probes were used to detect the expression of Sonic hedgehog (*Shh*), Dentin Sialophosphoprotein (*Dspp*), Ameloblastin (*Ambn*), *Axin2*, Fibroblast growth factor 10 (*Fgf10*) (33) and *Gli1* (mice, n = 3).

### Micro-Computed Tomography

Micro-computed tomography (μCT) was performed using a μCT 35 Scanner (Scanco Medical). Complete mandibular bones (mice, n = 6) were scanned with an isotropic voxel size of 12 μm using 70 kVp tube voltage, 114 μA tube current, 400 ms integration time, segmentation support of 1, and a sigma correction of 0.8. For the enamel reconstruction of 6 and 12 month old mice a threshold of 27% for dentin and 60% for enamel was chosen. In 1 month old animals the threshold for dentin was selected at 23% for dentin and 60% for enamel, respectively.

### Flow Cytometry

Isolated pulp tissue from 1 month old mice (mice, n = 6) was digested with 2 mg Collagenase type 1 (Worthington) and 4 mg Dispase II (Roche) in DMEM/F12 10% FCS for 1h at 37°C and constant rotation. The cells were pelleted at 2000 rpm (400g) for 5 min, resuspended in FACS staining buffer (5% FCS in PBS), incubated with the following prediluted antibodies at 1:100: CD45.2, Sca-1, CD90.2 (all Biolegend), and stained with Sytox Blue (Thermo Fisher Scientific). Data were collected on a FACScanto instrument (BD Biosciences) and analyzed using FlowJo software (TreeStar).

### Statistics

The data are shown as mean ± SD. Statistical analysis was performed using GraphPad Prism 5 software. Two-tailed, paired *t*-test was used for experiments involving two groups; p values lower than 0.05 were considered significant.

## Results

**Figure 2:**
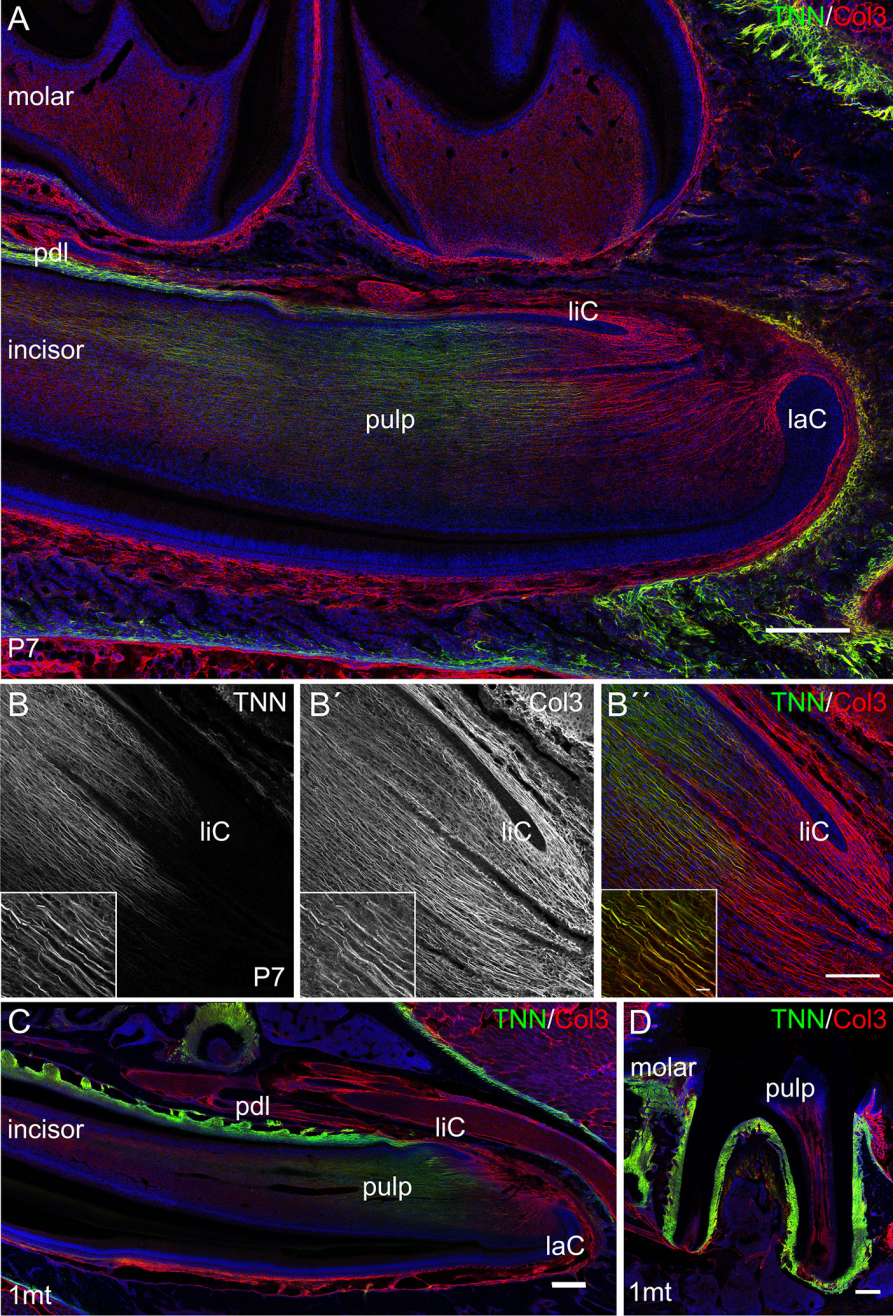
Expression of tenascin-W/TNN in the lower incisor pulp and periodontal ligament. **(A)** In longitudinal sections, tenascin-W/TNN staining (tenascin-W/TNN in green, collagen 3 (Col3) in red) is found in the distal pulp mesenchyme and in the periodontal ligament of one week old mice. However, no expression is detected in the most apical pulp, which represents the mesenchymal stem cell niche. **(B)** In the pulp tenascin-W/TNN (B TNN in grayscale and B″ TNN in green) immunostaining co-localizes with a thick parallel fiber network, which consists of collagen 3 containing fibers (B´ Col3 in grayscale and B″ Col3 in red). In adult molars **(C)** and incisors **(D)** tenascin-W/TNN is predominantly found in the periodontal ligament, in the incisor pulp the signal intensities for both collagen 3 and tenascin-W/TNN were decreased compared to perinatal incisors (laC labial cervical loop, liC lingual cervical loop, pdl periodontal ligament, mice, n = 3, scale bar 100 μm, magnified inlet 10 µm).

First, we analyzed the expression of tenascin-W/TNN in the incisor pulp mesenchyme during tooth development, using immunofluorescence. The expression started at the bell stage of tooth development, marked by ameloblast and odontoblast differentiation (34) (**Supplementary Figures 1A, B**). Tenascin-W/TNN is mainly found in the distal part of the dental papilla and colocalizes with thick spiral fibers (**Figure 2A**), while it is not detected in the apical part. Co-immunostainings showed that the tenascin-W/TNN labeled fibers contain collagen 3 (**Figures 2B, B´, B´´**). In newborn mice, tenascin-W/TNN specific immunostaining is concentrated in the area of pre-odontoblasts and mature odontoblasts (**Supplementary Figure 1C**). In adult mice, only a weak signal for tenascin-W/TNN is persistent in the distal pulp (**Figure 2C**); in contrast however tenascin-W/TNN is strongly expressed in the periodontal ligament of incisors and molars (**Figures 2C, D**).

**Figure 3:**
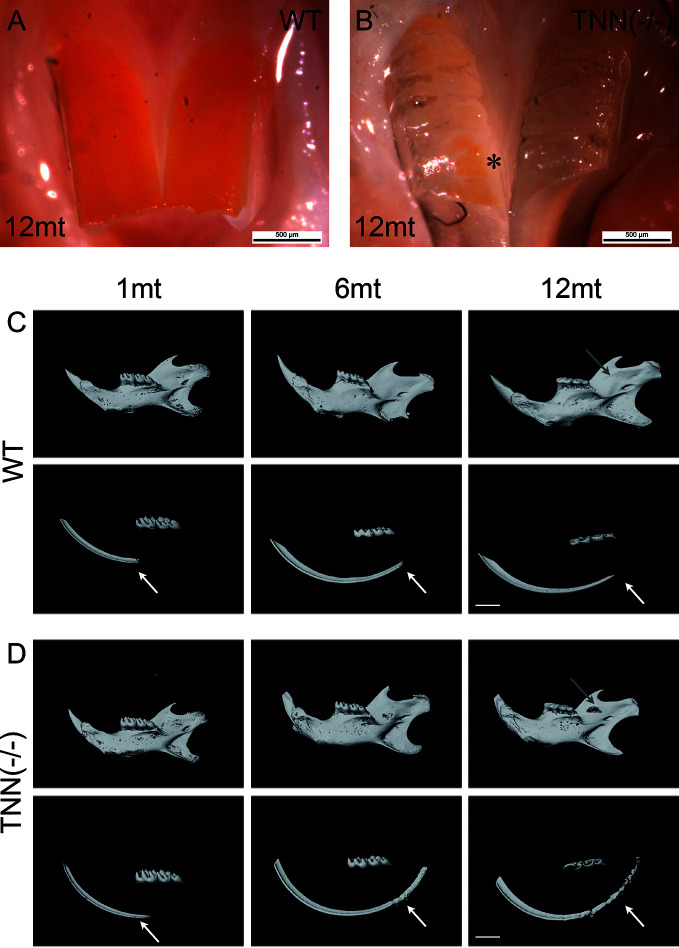
Macroscopic phenotype **(A, B)** and µCT analysis **(C, D)** of tenascin-W/TNN deficient mouse incisors. tenascin-W/TNN knockout mice develop normally, but show enamel defects in the upper incisor at the age of 6 months. The enamel of rodents contains iron ions and is therefore yellowish. Whereas in the wildtype **(A)** the entire buccal incisor surface is covered by enamel, in TNN(-/-) animals **(B)** only few enamel spots remain (asterisk). **(C, D)** Bone and dentin (upper panels) or dentin alone (lower panels) were imaged in the lower jaw by µCT. Compared to wildtype **(C)**, aged TNN(-/-) mice **(D)** are showing flatter lower incisor tips and a lingual bone fenestration in the incisor apical region (gray arrow). The 3D reconstruction of the enamel (**C, D**; lower panels) shows a dorsal shift of enamel mineralization toward the cervical loop (shown by an arrow) and a fragmented dorsal enamel layer (mice, n = 6, scale bar μCT: 2 mm).

To study the *in vivo* function of tenascin-W/TNN, we generated a global tenascin-W/TNN knockout mouse line. These mice are vital and fertile, but show an increased death rate and stress intolerance. Otherwise, tenascin-W/TNN mice develop and grow normally, with the exception of visible enamel defects in the upper incisors at 12 months (**Figures 3A, B**). To determine whether the lower incisors are affected as well, we analyzed lower jaws by micro-computed tomography (µCT). There were no obvious macroscopic differences in 1 month old animals, but 6 and 12 month old tenascin-W/TNN deficient mice showed a flattening of the incisor tip, suggesting a defect in abrasion and self-sharpening and/or altered occlusion. In addition, the mandibular bone is fenestrated in 12 month old TNN(-/-) mice in the area of the apical region of the incisor (**Figures 3C, D**, upper panel, gray arrow). By 3D reconstruction of the opaque enamel (**Figures 3C, D**, lower panel), we could observe that in tenascin-W/TNN deficient animals the enamel mineralization is shifted to the apical part of the incisor. Furthermore the apical enamel layer of 6 and 12 month old TNN(-/-) animals is fragmented (**Figures 3C, D**, lower panel).

**Figure 4:**
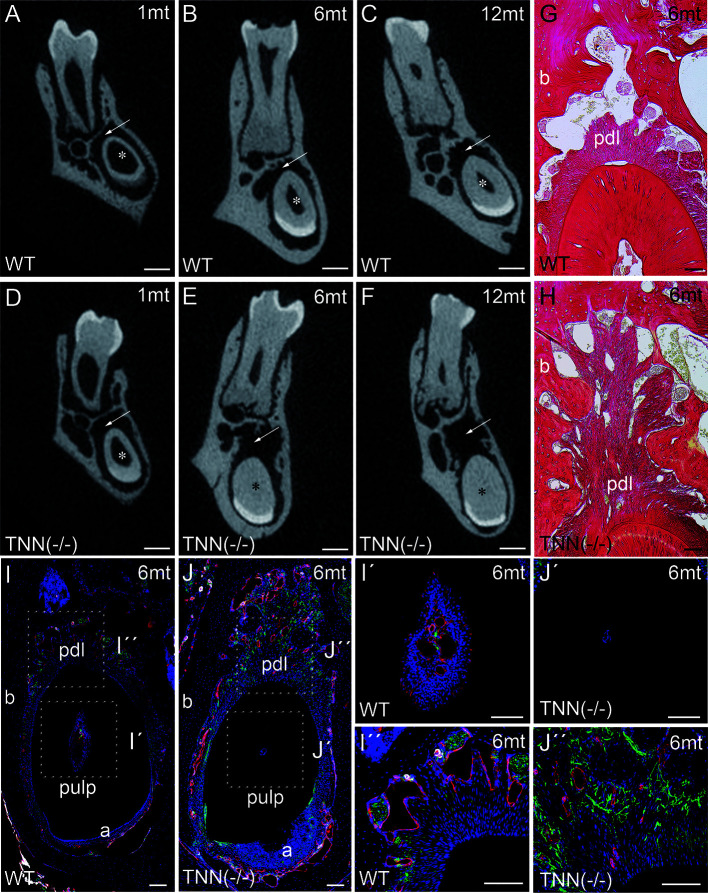
Analysis of lower incisor cross-sections **(A–F)** μCT scans at the heights of the first molar showed that compared to wildtype **(A–C)** the incisor dentin layer is much thicker in 1 month old tenascin-W/TNN deficient mice (**D**, asterisk), in 6 and 12 month old mice the incisor pulp is completely obliterated (**E**, **F**, asterisk). A second phenotypic change in the mutant is the wider incisor periodontal space, indicated by an arrow **(D–F)**. However, the periodontal space of the molars seems normal. **(G, H)** Sirius red stainings showed a significant alveolar bone loss and a replacement by a dense connective tissue in the mutant compared to wildtype. **(I, J)** An analysis of blood vessels (CD31 in red, smooth muscle actin (SMA) in white, Tuj1 in green) and nerve fibers revealed that in TNN-deficient mice, the remaining pulp tissue does not containing any projections **(I´, J´)**; in contrast the bone replacement tissue contains a well developed network of nerve fibers **(I´´,J´´)** (a ameloblasts; pdl periodontal ligament; mice, n = 6, scale bar μCT: 0.5 mm, histology: 100 μm).

μCT of lower incisors on the height of the first molar showed that the width of the dentin was increased in 1 month old TNN(-/-) animals (**Figures 4A, D**, asterisk), and in 6 and 12 month old animals the pulp space was completely obliterated (**Figures 4B, E** and **C, F**, asterisk). In addition, the periodontal space of the 6 and 12 month old tenascin-W/TNN knockout incisors was much wider (**Figures 4B, E**, and **C, F** arrow), while it was unchanged in the first molars (**Figures 4B, E**, and **C, F**). Histological analyses has shown a dense connective tissue of the periodontal ligament within a wider periodontal space of the mutant incisors (**Figures 4G, H**, pdl). Immunofluorescence stainings for nerve fibers (Tuj1), perivascular smooth muscle cells (smooth muscle actin), and endothelial cells (CD31) revealed that blood vessels and nerve fibers disappear in the distal pulp of tenascin-W/TNN knockout mice and that there is major loss in cellularity (**Figures 4I, J, I´, J´**). Furthermore, we have found a thick nerve fiber network within the periodontal ligament (**Figures 4I, J, I´´, J´´**).

**Figure 5:**
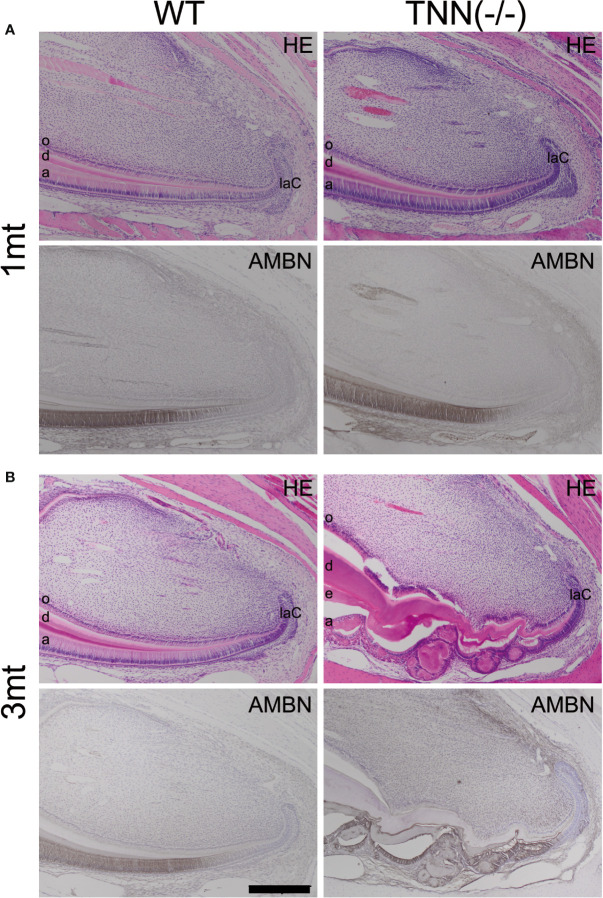
H&E and ameloblastin stainings of the apical region of the lower incisor The pulp was longitudinally sectioned at the height of the central arterioles. **(A)** In 1 month old tenascin-W/TNN deficient mice the width of the dentin layer is increased. **(B)** Severe epithelial and mesenchymal differentiation defects are visible in the apical region of 3 month old TNN(-/-) mice. Dense eosinophilic inclusions are found in the enamel epithelium. Immunohistochemical stainings with ameloblastin antibodies (black staining) showed that those inclusions contain enamel matrix proteins (a ameloblast, laC labial cervical loop, d dentin, e enamel, o odontoblasts, mice, n = 6, scale bar 200 μm).

The absence of tenascin-W/TNN expression at the level of the first molar in month old TNN-/- incisors (**Supplementary Figure 1E**) indicates that the changes in dentin thickness observed at this stage must be due to a secondary effect. Next, we analyzed the morphology of the stem cell niches and no morphological changes were observed in 1 month old mice (**Figure 5A**). In 3 month old TNN(-/-) mice both the dentin and enamel formation have increased and the enamel epithelium contains cyst-like structures (**Figure 5B**). Immunohistochemical staining for ameloblastin revealed that those epithelial inclusions are composed of enamel matrix (**Figure 5B**). Next, we analyzed the position of pre-ameloblasts and mature ameloblasts, and of odontoblasts in lower incisors. In 1 month old TNN(-/-) mice dentin sialophosphoprotein (*Dspp*), sonic hedgehog (*Shh*), and ameloblastin (*Ambn*) were expressed normally as determined by *in situ* hybridization. In 3 month old mice the differentiation of both ameloblasts (*Ambn* probe) and odontoblasts (*Dspp* probe) was shifted to the apex (**Supplementary Figure 2**). Stainings for endogenous alkaline phosphatase further confirmed this shift in differentiation (**Supplementary Figure 5**), and revealed that alkaline phosphatase activity is high in 1 year old TNN(-/-) mice (**Supplementary Figure 3**).

These findings indicate either a defect in growth factor signaling or a reduced number of stem cells in TNN-deficient incisors. We therefore performed *in situ* hybridizations with *Axin2*, *Fgf10*, and *Gli1* probes (**Supplementary Figure 4**). Expression of *Axin2*, a target and negative regulator of Wnt signaling, was chosen to analyze Wnt pathway activity, which controls dentin formation. FGF10 is produced in the pulp mesenchyme and maintains epithelial stem cells. Gli1 is a transcription factor that is expressed at sites of highly active hedgehog signaling (27). We found that tenascin-W/TNN deficiency leads to reduced expression of these three factors, indicating compromised Wnt, FGF, and hedgehog signaling in the region of the mesenchymal stem cell niche of the incisors, or, alternatively, loss of specific cell stem cell populations.

**Figure 6:**
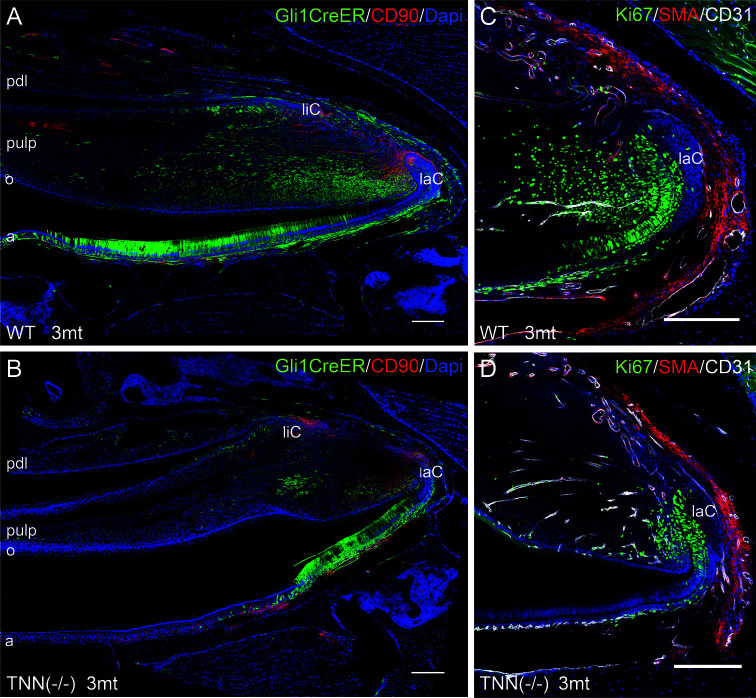
Differentiation and proliferation defects in the TNN-/- incisor stem cell compartment **(A, B)** Gli1 lineage tracing experiments in 3mt old animals. Gli1CreER^T2^ x R26mTmG x TNN(-/-) and Gli1CreER^T2^ x R26mTmG control mice were i.p. injected twice with 10 mg tamoxifen. The animals were sacrified 72 h after the first injection. **(A, B)** Sagittal sections of the incisor pulp reveal that the GFP marked Gli1 (in green) expressing cells are reduced in number and in area. The number of CD90.2 (in red) stem cell was not affected by tenascin-W/TNN deficiency. **(C, D)** tenascin-W/TNN deficient mice have less proliferative cells (Ki67 in green) in the stem cell compartment. The blood vessels (CD31 in green, smooth muscle actin (SMA) in white) are not affected by tenascin deficiency. (a ameloblasts, laC labial cervical loop, liC lingual cervical loop, o odontoblasts, pdl periodontal ligament, mice, n = 6, scale bar 200 μm).

We next performed lineage tracing experiments in Gli1CreER^T2^ x R26mTmG x TNN(-/-) and Gli1CreER^T2^ x R26mTmG control mice. 72 hour chase experiments showed that the pulp mesenchyme of 3 month old tenascin-W/TNN knockout mice contained only few Gli1 expressing GFP positive cells (**Figures 6A, B**) compared to controls. Ki67 immunostainings showed that the proliferation rate of putative mesenchymal stem cells was reduced in the incisors of 3 month old TNN(-/-) mice (**Figures 6C, D**).

These findings led to the question whether the mesenchymal stem cell themselves were affected or not. To resolve this issue, we performed a flow cytometry analysis of isolated apical pulp cells with CD45.2 [a general immune cell marker (35)], CD90.2(Thy1) [a marker for immune cells and incisor mesenchymal progenitor cells (3)], and Sca1 [a general stem and progenitor cell marker (36)] antibodies. CD45.2 was used to subtract the CD45.2/CD90.2 double positive immune cells, as described in Balic and Mina (37). This experiment showed no differences in the number of CD90.2 or Sca1 positive progenitor cells in 1 month old control or knockout animals (**Supplementary Figure 5**). Furthermore, we performed immunostainings with a CD90.2 and Sox10 antibodies. Sox10 is a marker for pluripotent neural crest cells and glia cells (38) and was used to identify nerve-derived mesenchymal stem cells in the incisor (3). The stainings showed an accumulation of Sox10 positive cells in the apical pulp mesenchyme of 3 mt old tenascin-W/TNN deficient animals; however, CD90.2 immunolocalization was not altered. This indicates that the nerve-derived mesenchymal stem cells of TNN knockout incisors become blocked in their differentiation pathway and accumulate (**Supplementary Figure 6**).

**Figure 7:**
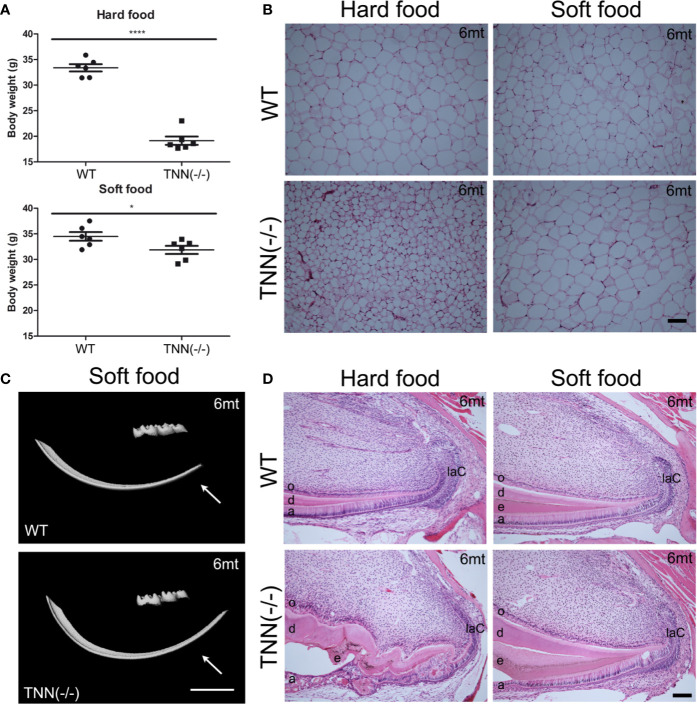
Soft food diet ameliorates the TNN-/- incisor phenotype **(A)** Soft food diet decreases the weight loss observed in aged tenascin-W/TNN knockout mice. **(B)** tenascin-W/TNN knockout mice fed with hard food diet have significantly less white adipose tissue, soft food diet equilibrates the amount of epigonadal white adipose tissue. **(C)** The enamel layer of soft food diet fed TNN(-/-) mice is continuous. **(D)** Histological analysis of the apical region shows a significant rescue on the cellular level. (a ameloblasts, laC labial cervical loop, d dentin, e enamel, o odontoblasts, mice, n = 6, scale bar μCT: 2 mm, histology: 100 μm).

**Figure 8:**
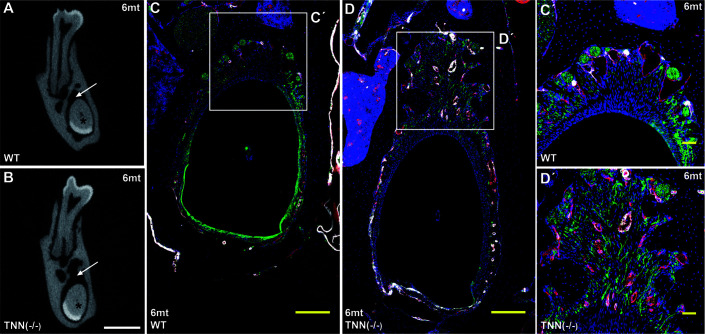
Soft food diet does not revert the TNN-/- periodontal defect μCT cross-sectional scans at the height of the first molar showed incisor pulp obliteration in both 6 month old wildtype and tenascin-W/TNN deficient mice (**A, B**, asterisk). The wider periodontal space, indicated by an arrow (A and B, arrow) is still found in soft diet fed 6 month old tenascin-W/TNN deficient mice. Immunofluorescence stainings **(C´, D´)** confirmed the presence of branched nerve fibers (CD31 in red, smooth muscle actin (SMA) in white, Tuj1 in green) in tenascin-W/TNN deficient periodontal space. (scale bar μCT: 1 mm, mice, n = 6, histology: **(C, D)** 200 μm, **(C´, D´)** 50 μm).

Interestingly, aged TNN(-/-) mice never develop obesity. The body weight of 1 year old knockout animals was in both sexes significantly reduced (**Figure 7A**). We hypothesized that TNN(-/-) induced damage and tooth pain prevent proper food intake. To test this hypothesis we fed our mice with a soft food diet, which showed that the physical form of the diet was sufficient to rescue the body weight. Hence, the malformed teeth are the cause for the observed weight differences (**Figures 7A, B**). The weight of the wild type mice did not change with a soft diet. Soft food also ameliorated the incisor phenotype of knockout mice: μCT reconstructions of the enamel layer showed that the enamel layer remained continuous (**Figure 7C**), and histological sections of the incisor apical region showed improved epithelial and mesenchymal differentiation (**Figure 7D**). Next, we analyzed μCT cross sections of soft food fed 6 month old mice (**Figures 8A, B**). The pulp was now obliterated both in wild type and TNN deficient mice, indicating a slower tooth growth rate under soft food diet. Interestingly, the alveolar bone loss of TNN deficient mice persisted as well as the increased nerve branching under soft food diet (**Figures 8C, D**). This finding demonstrates that the periodontal ligament defect is the primary cause of the incisor eruption phenotype.

## Discussion

Our data reveal that tenascin-W/TNN is distinctly localized in the extracellular matrix and partially co-localizes with collagen 3 fibers. In teeth, this distribution pattern is different from tenascin-C, which is more diffusely distributed in the connective tissues. Earlier studies on tenascin-W/TNN have mostly focused on the *in vitro* function or tissue expression in cancer (23, 24). Here, we report the first tenascin-W/TNN deficient mouse for functional studies.

The most striking phenotype of tenascin-W/TNN deficient mice is found in the incisors, which show irregular dentin and enamel formation, while no phenotype changes are seen in molars. Furthermore, we have observed alveolar bone loss and increased nerve branching in the significantly wider periodontal ligament of TNN deficient mice. Changes in the periodontal ligament observed in the TNN-/- mice suggest that connective tissue remodeling and continuous eruption are disturbed. Initial tooth eruption in rodents relies heavily on monocyte/macrophage lineage cells as demonstrated by toothless rats (39) and in Csf1 deficient mice neither molar nor incisors erupt (40). However, little is known about the role of monocyte/macrophage lineage cells in continuous incisor eruption. The periodontal ligament is very different from the dental sac which is responsible for initial tooth eruption. Tenascins are known to support cell migration *in vitro* (23) and to modulate macrophage activity by TLR4 binding (41). These known functions are suggesting that in TNN-deficient mice remodeling of the periodontal ligament might be affected through altered macrophage migration or activity. Another hypothesis is that tenascin-W/TNN affects the mechanical properties of the periodontal ligament and periodontal fibroblasts are differently regulated. Changes in the connective tissue stiffness are known to modulate extracellular matrix remodeling (42). Tenascin-X knockout mice have altered collagen fibers mimicking Ehlers-Danlos syndrome (20). However, in tenascin-W/TNN deficient mice ultrastructural analysis of the periodontal ligament showed normal collagen fibers (data not shown). Furthermore, we have observed alveolar bone loss and increased nerve branching in the periodontal ligament of TNN deficient mice. Since the rodent incisor periodontal ligament is rich in Ruffini- and free nerve endings (43), it is likely that this defect leads to pain or discomfort. In agreement with this assumption we found in aged tenascin-W/TNN deficient mice a significant reduction of the body weight.

Since the eruption rate of incisors is under neural and mechanical control (44) we wondered whether soft food diet accompanied with wooden gnaw sticks would rescue the body weight defect. We found that the TNN deficient mice gained weight comparably to control mice under these conditions, and the irregular dentin and enamel formation of the incisor disappeared. However, the periodontal defect including alveolar bone loss remained in the soft food treated TNN deficient animals. This important finding indicates that the odontoblast and ameloblast anomalies observed in TNN deficient incisors under standard hard food diet are secondary to a primary defect in the periodontium, and might be caused by mechanical damage to the pulp and/or pain-related processes.

Next, we examined the impact of tenascin-W/TNN deficiency on the incisor stem cell niche under standard food diet. The decreased Gli1 expression and cell proliferation suggests reduced hedgehog activity (4), and again indicate that the irregular tooth formation is of secondary nature, as tenascin-W/TNN is not expressed in the stem cell niche and the percentage of progenitor cells is not altered. Hendaoui et al. (45) proposed that tenascin-C and –W/N modulate Wnt signaling by sequestering the growth factor. However, tenascin-W/TNN is not expressed in the incisor stem cell niche itself and therefore cannot modulate growth factor signaling directly. Hence, axin2 and Gli1 expression and cell proliferation are clearly due to secondary effects. Since it is very difficult obtaining comparable sections, we decided to include only unquantified data.

Similar phenotypes in the incisors have been observed in periostin or integrin α11 deficient mouse lines; both proteins play central roles in periodontal ligament remodeling and mechanosignaling. One of them, periostin, is a characteristic extracellular matrix component of the periodontal ligament, and periostin deficiency in mice leads to periodontal defects in incisors and molars. Periostin has a known function in collagen 1 fibrillogenesis (46) by supporting proteolytic activation of lysyl oxidase (47). Furthermore, periostin plays a role in vascular smooth muscle cell migration (48) and in the migration of bone lining cells (49). Interestingly, soft chow also ameliorates the phenotype observed in periostin deficient animals (50). One major difference to the phenotype observed in TNN knockout mice is that periostin deficiency affects also the molar periodontal ligament (50), not just that of the continuously erupting incisor. The only other extracellular matrix related knockout where the incisors are affected is that of integrin α11 (51). Integrin α11β1 functions as a collagen receptor and it has been suggested that it plays a role in mechanotransduction. Interestingly, soft food diet ameliorates also the integrin α11 phenotype (51). Both periostin and integrin α11 play a role in the remodeling of collagen 1 fibers. The irregular tooth formation observed in periostin deficient mice might be due to a periodontal ligament—neurosecretory feedback mechanism. Based on published data (41), we hypothesize that tenascin-W/TNN acts on macrophages or fibroblasts directly, and that the underlying mechanism of the remodeling defect is different.

In conclusion, our data show that tenascin-W/TNN co-localizes with collagen 3 fibers in the pulp and the periodontal ligament. Here, we present the first characterization of tenascin-W/TNN deficient mice and we conclude that tenascin-W/TNN plays a pivotal role in incisor periodontal remodeling and diet uptake. The periodontal remodeling phenotype is furthermore connected with nerve branching. Since soft food diet results in equal body weights, but the periodontal defect persists in these mice, we assume that nerve branching might lead to pain. Since tenascin-W/TNN itself is not expressed in the stem cell compartment nor in the proliferation zone, we hypothesize that the effect in the pulp is of secondary nature, most probably due to reduced neural input. The diminished Gli1 expression in the pulp supports this assumption. Neuro-vascular secreted sonic hedgehog is a known regulator in this stem cell niche and Gli1 is the main effector of activated hedgehog signaling. In summary, we report for the first time about the *in vivo* function of tenascin-W/TNN and we show that this extracellular matrix protein plays a crucial role in periodontal remodeling.

## Data Availability Statement

The raw data supporting the conclusions of this article will be made available by the authors, without undue reservation.

## Ethics Statement

The animal study was reviewed and approved by Landesamt für Natur, Umwelt und Verbraucherschutz Nordrhein-Westfalen (LANUV NRW), Postfach 10 10 52, 45610 Recklinghausen.

## Author Contributions

Study design: TI. Animal treatment: TI. Histology, *in situ* hybridization, and immunostaining: TI, AB, IT. Tenascin W/TNN antibody production: RC-E. μCT analysis: JH and AN. FACS analysis: TI and BB. Data interpretation: TI, AB, IT, and MK. Manuscript preparation: TI, MC, and BB. All authors contributed to the article and approved the submitted version.

## Funding

This work was supported by grants from the DFG (A2, CRC829; B2, D1, M1 FOR2722) and Köln Fortune to MK. the DFG (A2, CRC829; B2, D1, M1 FOR2722) and Köln Fortune to MK.

## Conflict of Interest

The authors declare that the research was conducted in the absence of any commercial or financial relationships that could be construed as a potential conflict of interest.

## Supplementary Material

Supplementary Figure 1
**(A, B)** Late expression of tenascin-W/TNN in the incisor pulp and tenascin-W/TNN expression in cross-sections Until the early bell stage (**A**; E16) no tenascin-W/TNN (TNN in green) is expressed in the incisor pulp. Blood vessels (CD31 in red) were stained to easily distinguish the mesenchyme from the epithelium. At the late bell stage almost the entire distal pulp mesenchyme stains for tenascin-W/TNN (**B**; E19). However, tenascin-W/TNN is not expressed in the mesenchymal stem cell niche. **(C)** tenascin-W/TNN (TNN in green, CD31 in red) is strongly detected in the odontoblast layer of newborn mice. tenascin-W/TNN stains a thick spiral-shaped fiber network. **(D)** The immunostaining for tenascin-W/TNN is highly specific, in TNN(-/-) mice no signal for tenascin-W/TNN is found. (**E,E**’) tenascin-W/TNN (TNN in green) is not detected in the distal incisor pulp of 1 month old mice. In the periodontal ligament tenascin-W/TNN colocalizes with collagen 3 (Collagen (Col3) in red) (liC lingual cervical loop, laC labial cervical loop, mice, n = 3, scale bar **(A, B)** 200 μm).

Supplementary Figure 2
*In situ* hybridization for differentiation markers Differentiation of ameloblasts and odontoblasts was further analyzed by *in situ* hybridization experiments with probes for ameloblastin (*Ambn)*, dentin sialophosphoprotein (*Dspp)*, and sonic hedgehog (*Shh)*. **(A)** Schematic overview of the incisor epithelial cell layers. Ameloblastin is expressed in pre-odontoblasts, pre-ameloblasts, and ameloblasts. Dentin sialophosphoprotein is expressed in pre-odontoblasts and odontoblasts, sonic hedgehog is expressed in pre-ameloblasts and in the stratum intermedium of the enamel organ. **(B, C)** In 1 month old mice we found no differences in the expression of differentiation markers. In 3 month old TNN-deficient mice the cells differentiate closer to the cervical loop (**C**, **D** arrow) and the cervical loop seems smaller (*Ambn* ameloblastin, *Shh* sonic hedgehog, *Dspp* dentin sialophosphoprotein, a ameloblasts, laC labial cervical loop, o odontoblasts, mice, n = 3, scale bar 100 μm).

Supplementary Figure 3 Alkaline phosphatase staining. The epithelial and mesenchymal differentiation was analyzed by staining for endogenous alkaline phosphatase. Differentiating odontoblasts are detected in the pulp of 1 year old TNN(-/-) mice. The signal in the pre-odontoblast layer is shifted to the cervical loop region (arrow) indicating earlier differentiation. (a ameloblasts, laC labial cervical loop, o odontoblasts, mice, n = 6, scale bar 200 μm).

Supplementary Figure 4 Defective FGF, Wnt, and Shh signaling in TNN (-/-) mice **(A–F)** *In situ* hybridization experiments for *Axin2* **(A, D)**, *Fgf10* **(B, E)**, and *Gli1* **(C, F)** showed reduced expression of these markers in the mesenchymal stem cell niche in 1 month old TNN-deficient **(D–F)** compared to wildtype **(A–C)** mice. (a ameloblasts, laC labial cervical loop, liC lingual cervical loop, o odontoblasts, pdl periodontal ligament, mice, n = 3, scale bar 200 μm).

Supplementary Figure 5
**(A)** Representative FACS analysis of isolated incisor pulp cells from wildtype **(A)** and TNN(-/-) **(B)** mice. Sytox Blue dead cell stain negative and CD45 negative cells were analyzed with Sca-1 and CD90.2 markers. **(C)** FACS analysis of 1 month old mice showed that the number of CD90.2 and Sca1 positive progenitor cells is unchanged (SSC-A Side Scatter A, FSC-A Forward Scatter A, mice, n = 6).

Supplementary Figure 6 Sox10 positive cells accumulate in the region of the neurovascular bundle **(A–H)** Cross sections of the apical region showed an accumulation of Sox10 (Sox10 in red, CD90 in white, Tuj1 in green) positive cells in the pulp of 3 month old tenascin-W/TNN knockout mice. In the knockout mice the number of Sox10 positive cells increased with ageing. **(I, J)** Sagittal sections showed that the pulp of 1 year old tenascin-W/TNN mice is full of Sox10 positive cells. CD90.2 positive cells are found in the most apical mesenchyme (a ameloblasts, laC labial cervical loop, liC lingual cervical loop, mice, n = 6, scale bar 200 μm).
