# Supplementary figures and images for: Pivotal Role of Tenascin-W (-N) in Postnatal Incisor Growth and Periodontal Ligament Remodeling
Source: Front Immunol. 2021 Jan 22;11:608223. doi: 10.3389/fimmu.2020.608223 (PMC7862723; doi:10.3389/fimmu.2020.608223)

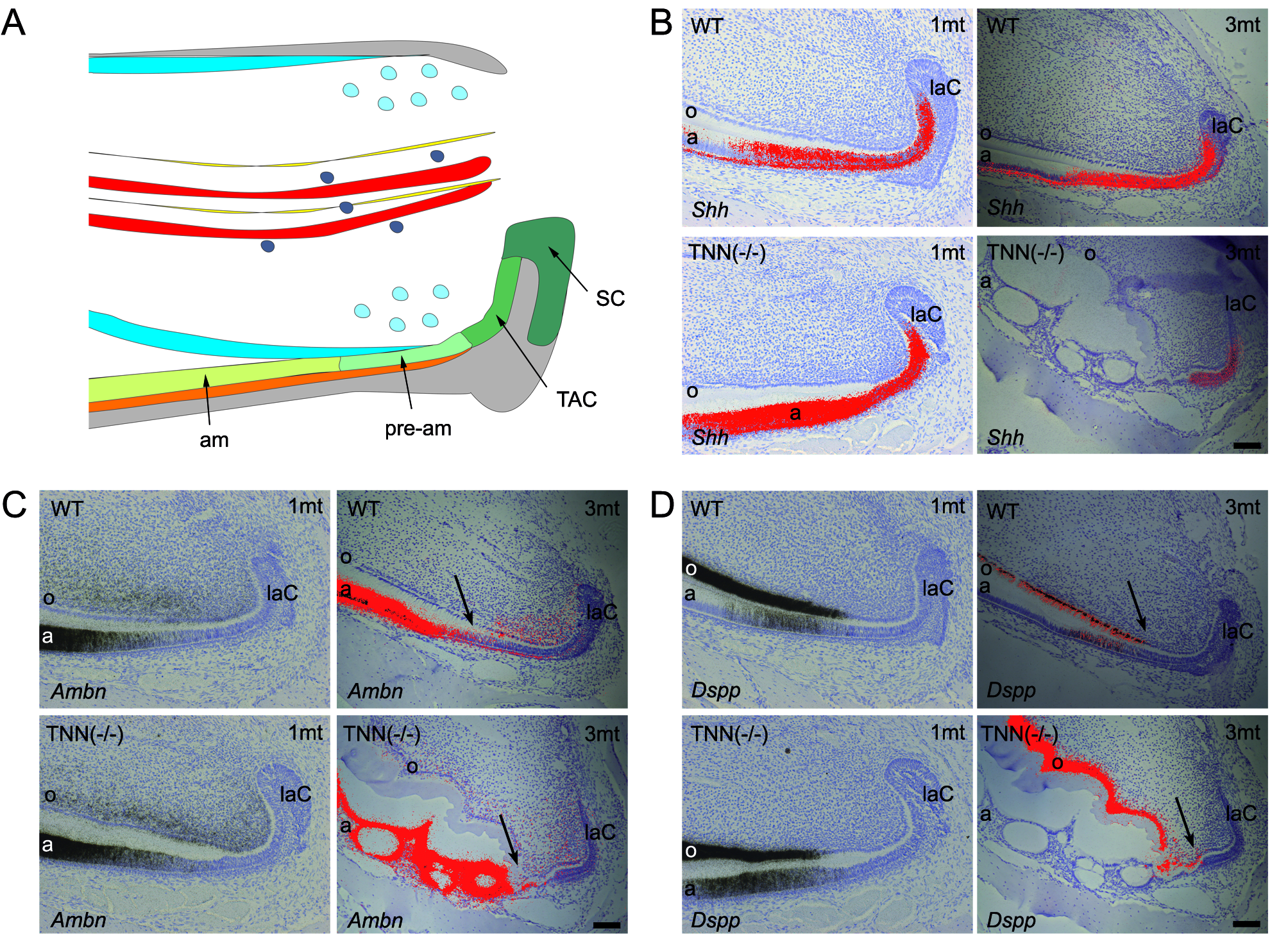

Supplement: Supplementary Figure 2 — In situ hybridization for differentiation markers Differentiation of ameloblasts and odontoblasts was further analyzed by in situ hybridization experiments with probes for ameloblastin (Ambn), dentin sialophosphoprotein (Dspp), and sonic hedgehog (Shh). (A) Schematic overview of the incisor epithelial cell layers. Ameloblastin is expressed in pre-odontoblasts, pre-ameloblasts, and ameloblasts. Dentin sialophosphoprotein is expressed in pre-odontoblasts and odontoblasts, sonic hedgehog is expressed in pre-ameloblasts and in the stratum intermedium of the enamel organ. (B, C) In 1 month old mice we found no differences in the expression of differentiation markers. In 3 month old TNN-deficient mice the cells differentiate closer to the cervical loop (C, D arrow) and the cervical loop seems smaller (Ambn ameloblastin, Shh sonic hedgehog, Dspp dentin sialophosphoprotein, a ameloblasts, laC labial cervical loop, o odontoblasts, mice, n = 3, scale bar 100 μm). [file Image_2.tif]

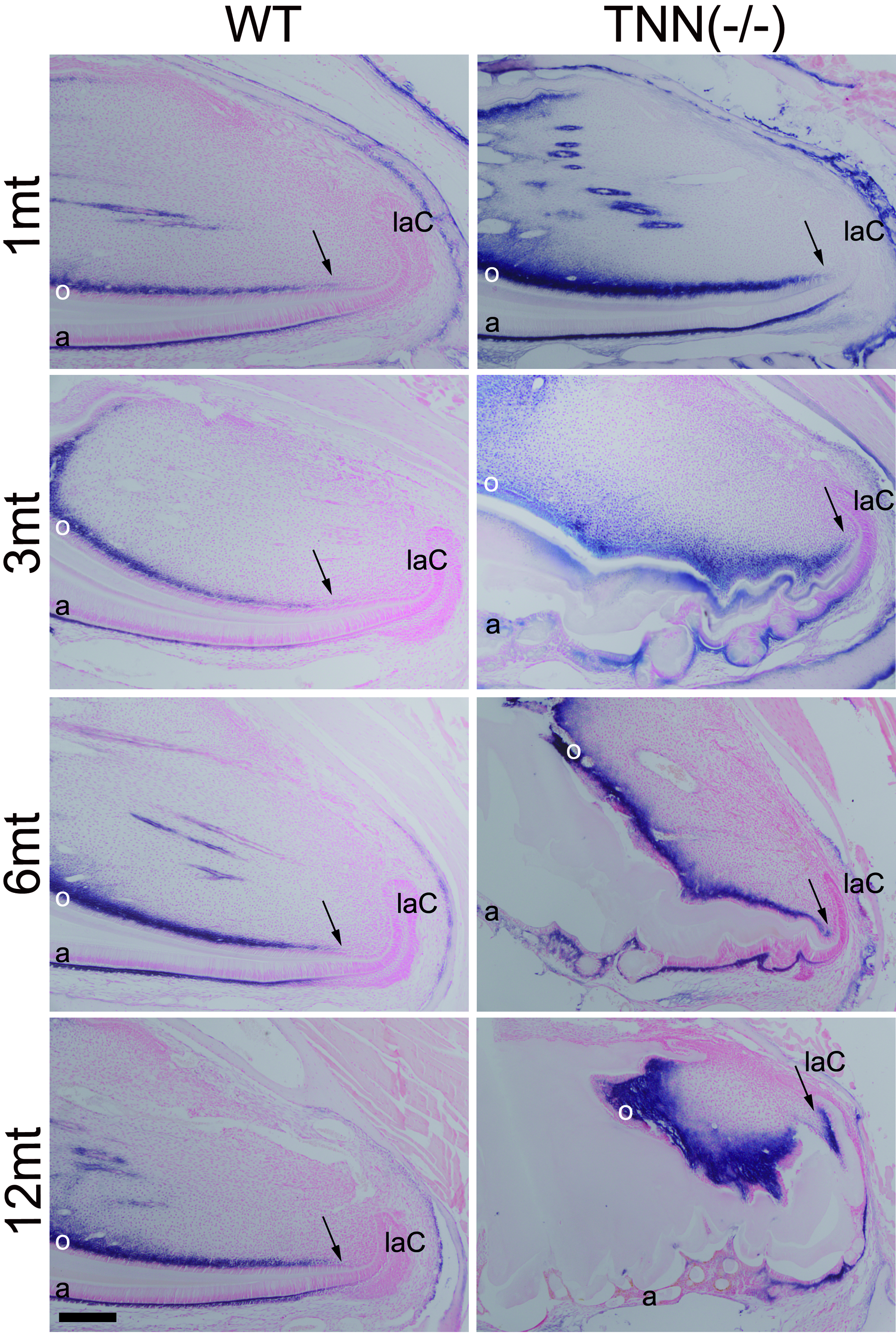

Supplement: Supplementary Figure 3 — Alkaline phosphatase staining. The epithelial and mesenchymal differentiation was analyzed by staining for endogenous alkaline phosphatase. Differentiating odontoblasts are detected in the pulp of 1 year old TNN(-/-) mice. The signal in the pre-odontoblast layer is shifted to the cervical loop region (arrow) indicating earlier differentiation. (a ameloblasts, laC labial cervical loop, o odontoblasts, mice, n = 6, scale bar 200 μm). [file Image_3.tif]

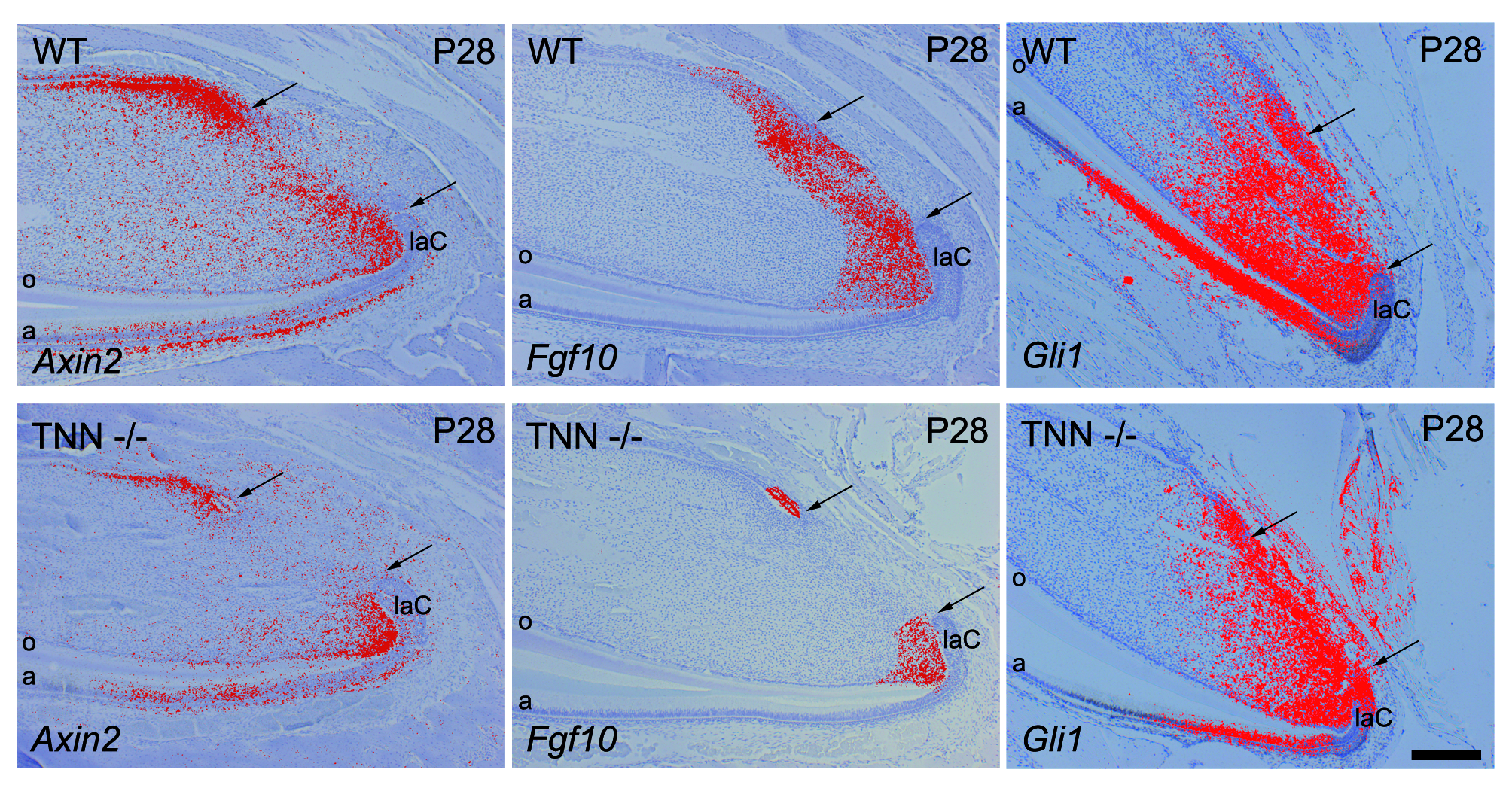

Supplement: Supplementary Figure 4 — Defective FGF, Wnt, and Shh signaling in TNN (-/-) mice (A–F) In situ hybridization experiments for Axin2 (A, D), Fgf10 (B, E), and Gli1 (C, F) showed reduced expression of these markers in the mesenchymal stem cell niche in 1 month old TNN-deficient (D–F) compared to wildtype (A–C) mice. (a ameloblasts, laC labial cervical loop, liC lingual cervical loop, o odontoblasts, pdl periodontal ligament, mice, n = 3, scale bar 200 μm). [file Image_4.tif]

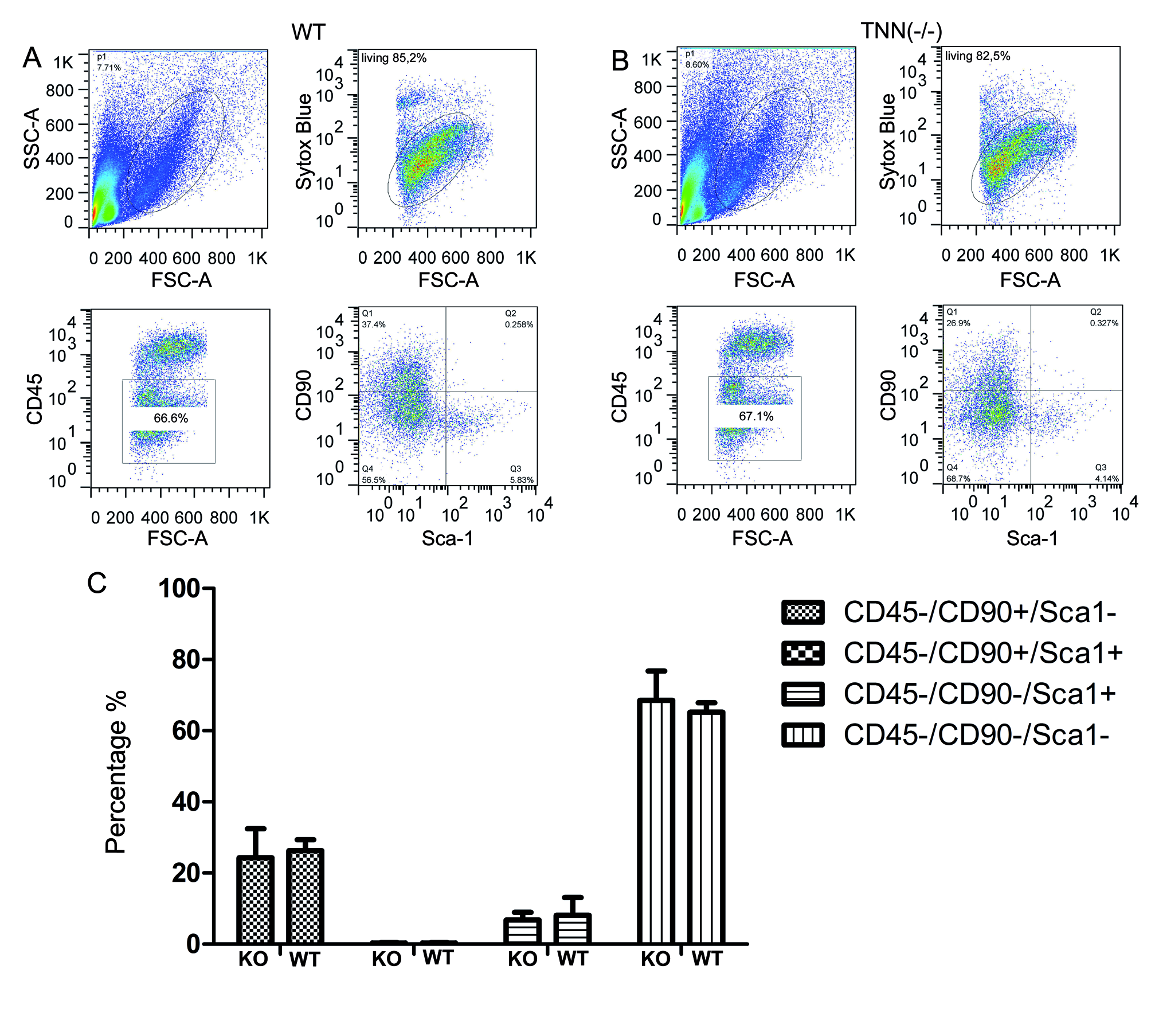

Supplement: Supplementary Figure 5 — (A) Representative FACS analysis of isolated incisor pulp cells from wildtype (A) and TNN(-/-) (B) mice. Sytox Blue dead cell stain negative and CD45 negative cells were analyzed with Sca-1 and CD90.2 markers. (C) FACS analysis of 1 month old mice showed that the number of CD90.2 and Sca1 positive progenitor cells is unchanged (SSC-A Side Scatter A, FSC-A Forward Scatter A, mice, n = 6). [file Image_5.tif]

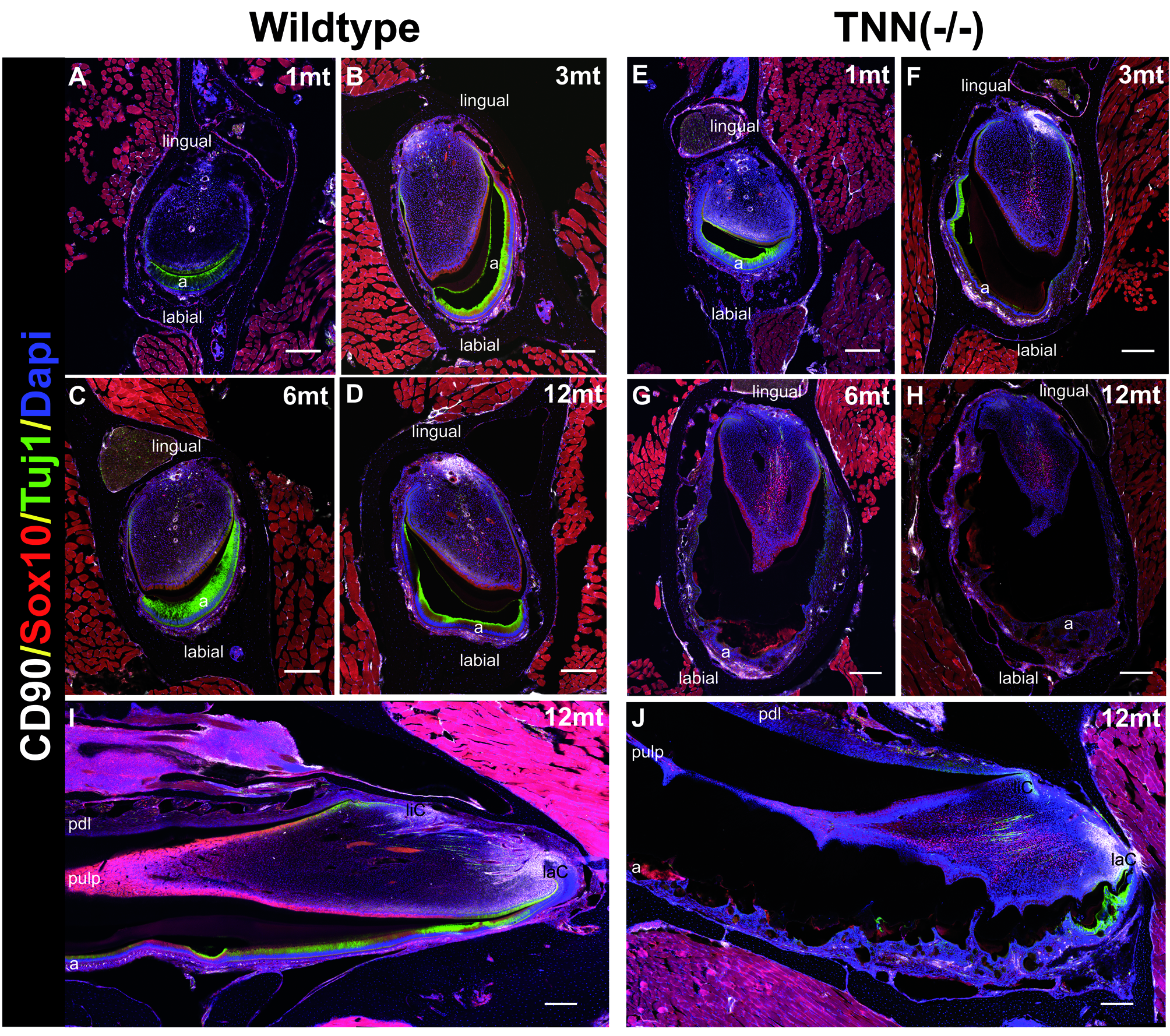

Supplement: Supplementary Figure 6 — Sox10 positive cells accumulate in the region of the neurovascular bundle (A–H) Cross sections of the apical region showed an accumulation of Sox10 (Sox10 in red, CD90 in white, Tuj1 in green) positive cells in the pulp of 3 month old tenascin-W/TNN knockout mice. In the knockout mice the number of Sox10 positive cells increased with ageing. (I, J) Sagittal sections showed that the pulp of 1 year old tenascin-W/TNN mice is full of Sox10 positive cells. CD90.2 positive cells are found in the most apical mesenchyme (a ameloblasts, laC labial cervical loop, liC lingual cervical loop, mice, n = 6, scale bar 200 μm). [file Image_6.tif]
